# An unusual case of Takayasu arteritis presenting as isolated severe aortic regurgitation: A case report

**DOI:** 10.1016/j.ijscr.2023.108407

**Published:** 2023-06-15

**Authors:** Ankita Simkhada, Pritha Acharya, Sansar Babu Tiwari, Bibek K.C.

**Affiliations:** a Department of Pathology, Institute of Medicine, Tribhuvan University Teaching Hospital, Maharajgunj, Kathmandu, Bagmati Province, Nepal; b Department of Pathology, Madan Bhandari Academy of Health Sciences, Hetauda, Bagmati Province, Nepal

**Keywords:** Aneurysm, Aortic regurgitation, Takayasu arteritis, Vasculitis, Case report

## Abstract

**Introduction:**

Takayasu arteritis is a chronic granulomatous vasculitis involving the large vessels, mainly the aorta and its branches.

**Case presentation:**

We report a case of a young female who presented with severe shortness of breath and easy fatigability. Examination revealed a diastolic murmur and imaging studies revealed a severe aortic regurgitation with aneurysm of the aortic root and ascending aorta.

**Clinical discussion:**

Modified Bentall's procedure was performed and sample sent for histopathology which showed granulomatous inflammation of the aorta with elastic fibre destruction in the medial layer. Infective causes of aortic aneurysm were ruled out and a diagnosis of Takayasu arteritis was made on the basis of clinical, radiological and histological findings.

**Conclusion:**

This case highlights the unusual presentation of Takayasu arteritis in which the patient had severe aortic regurgitation and aneurysm of the ascending aorta without steno-occlusive lesion elsewhere.

## Highlights

• Takayasu arteritis is a large vessel vasculitis.
• Affects young adults in the second or third decade with a conspicuous female preponderance
• Histopathology shows loss of elastic fibres in the intima media.
• Often found in association with steno occlusive lesions and isolated aneurysm of aorta is rare
• Treatment is primarily immunosuppressants, with some cases requiring surgical intervention.

## 1 Introduction

Takayasu arteritis (TA) is an idiopathic large vessel vasculitis with propensity for aorta and its branches. Cases of TA have been reported worldwide with an estimated incidence of 1.11 per million person-years [1]. The incidence is higher in Southeast Asia, Central and South America and Africa [2]. TA affects young adults in the second or third decade with a conspicuous female preponderance [3].

Inflammation of large vessels starts from the adventitia eventually progressing to pan-aortitis involving all layers of the vessel wall [3]. The inflammation and endothelial damage may lead to wall thickening and thrombus formation leading to lumen occlusion or stenosis of vessels wall [3]. On the other hand, destruction of the elastic fibres in the media leads to dilatation and aneurysm of the involved vessel [3,4].

Our case report illustrates an unusual presentation of Takayasu arteritis where the patient had severe aortic regurgitation and aneurysm of the ascending aorta without steno-occlusive lesion elsewhere.

This case report has been produced in keeping with the SCARE criteria [5].

## 2 Presentation of case

A 21 year female presented with chief complaints of shortness of breath, malaise, easy fatigability, recurrent severe headache and chest pain on exertion for the past year. She did not have any significant history of previous illnesses or chronic diseases. On examination, she had normal blood pressure and all peripheral pulses were palpable. A diastolic murmur was heard on auscultation. Relevant blood investigations were sent which revealed normal haemoglobin levels along with normal renal and liver function tests. Serological tests for HIV, hepatitis B/C and Syphilis were negative. ESR levels were raised to 90 mm/h and CRP was slightly raised to 25 mg/L. CT scan of the head showed mild cortical atrophy.

**Fig. 1:**
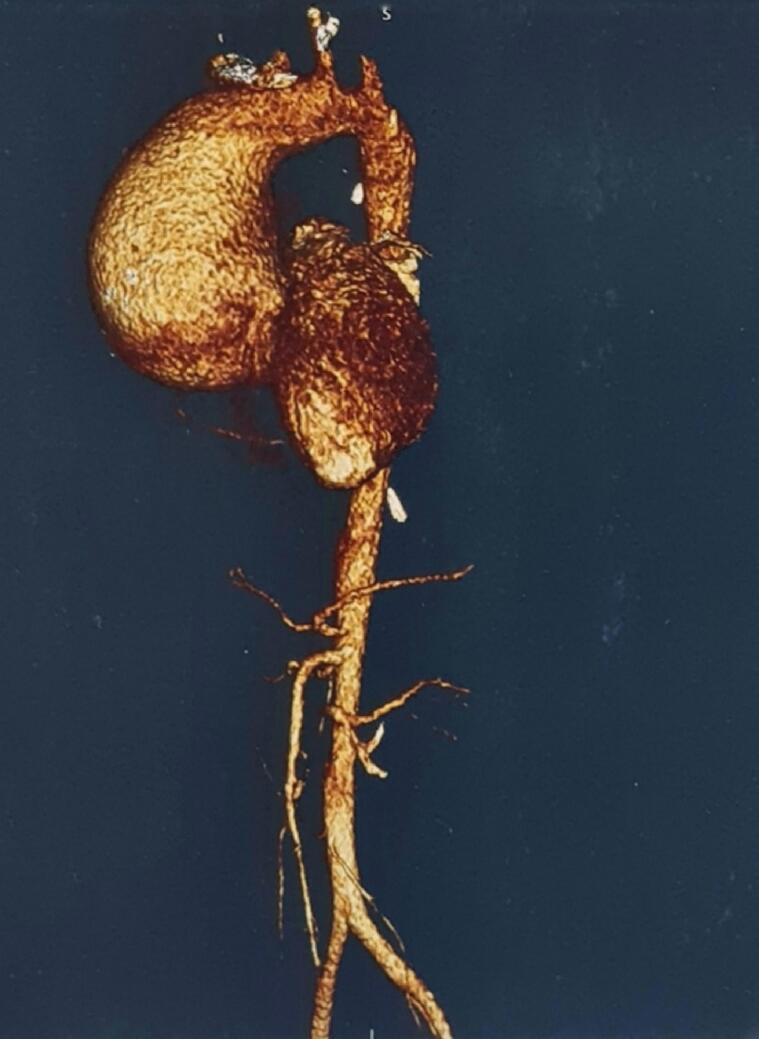
3D reconstruction CT angiography. Marked dilatation of the ascending aorta is noted.

**Fig. 2:**
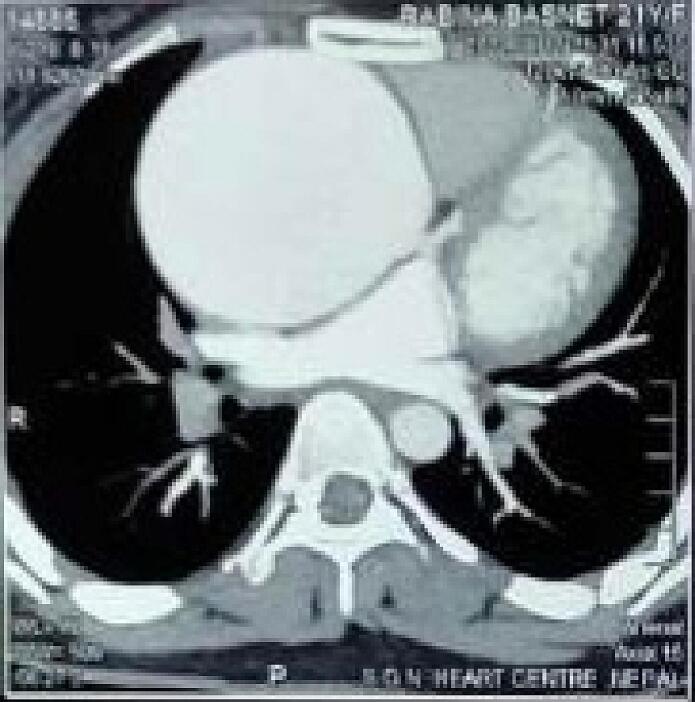
Computed tomography (CT) angiography. Axial view showing cross-section of the aneurysm at the level of ascending aorta.

Chest X-ray revealed widening of the aortic silhouette. Electrocardiogram showed a sinus rhythm with a heart rate of 66 beats per minute. Transthoracic echocardiography revealed severe aortic regurgitation with moderate mitral regurgitation and a dilated left ventricle (Left ventricular internal diameter: 6.5/4.7 cm) along with dilated aortic root and ascending aorta. Left ventricular ejection fraction was 50 %. Multidetector computed tomography of the chest showed aneurismal dilatation of the ascending aorta (maximum diameter 7.5 cm at the mid ascending aorta) with dilatation of the aortic root (Fig. 1, Fig. 2). Doppler study of the carotid and vertebral arteries showed normal blood flow with no intimo-medial thickening of the arteries.

**Fig. 3:**
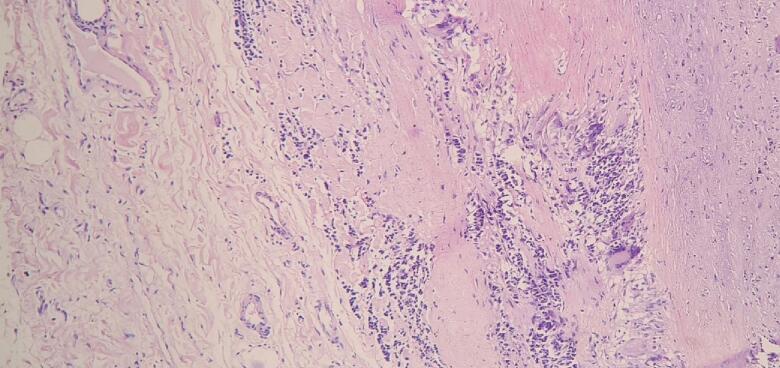
Histological finding in Takayasu arteritis. Intimal thickening, disruption of the elastic fibres in medial layer with few giant cells, fibrous expansion of the adventitia with inflammation surrounding the vasa vasorum (Haematoxylin and Eosin, ×100).

**Fig. 4:**
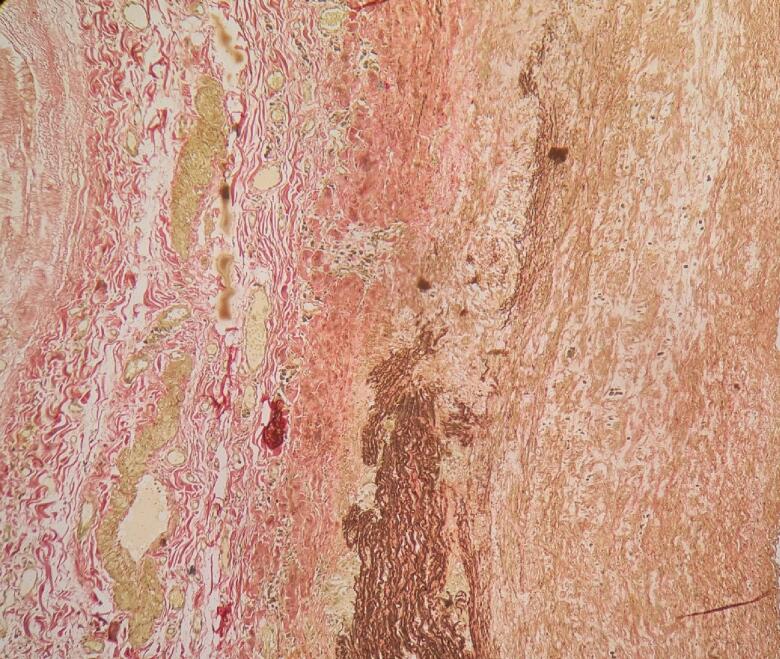
Takayasu arteritis. Elastic staining of the aorta showing intimal thickening, marked disruption of elastic fibres in the medial layer and adventitial fibrous thickening (Elastic van Gieson ×100).

She underwent Modified Bentall's procedure and the diseased dilated aorta was resected and sent for histopathological examination at our centre. Haematoxylin and Eosin stained sections of the aorta showed mononuclear cell infiltrates in the tunica media and adventitia with fragmentation of elastic fibres in along with necrosis and giant cell granulomatous reaction in the tunica media (Fig. 3). Van Gieson staining showed marked disruption of the elastic fibre in tunica media (Fig. 4). Ziehl Neelsen stain and PAS stain were negative for acid fast bacilli and fungal elements respectively.

Her postoperative period was uneventful and she was discharged 15 days after the surgery on a prescription of corticosteroids, antibiotics and anticoagulants. Four months post-surgery, she has been doing well with no fresh complaints.

## 3 Discussion

In 1908, a Japanese ophthalmologist, Mikito Takayasu, first described the retinal arterio-venous abnormalities in a patient with the disease after which the disease was named as such [6]. It is thought that an unknown stimulus leads to activation of heat shock protein 65 in the aorta. Activation of this protein in turn leads to expression of MHC I chain related protein A on vascular cells which is recognized by T lymphocytes and macrophages. These inflammatory cells are then stimulated to produce proinflammatory cytokines such as tumour necrosis factor (TNF) α and interferon (INF) γ resulting in inflammation, necrosis, neovascularisation, intimal proliferation and giant cell formation [3]. B lymphocytes producing autoantibodies against the endothelial cells are also activated, which further contribute to the inflammatory process [3,6]. The mycobacterial species has a 65 kDa heat shock protein homologous to that in humans. An immunologically mediated cross reaction can occur, leading to an autoimmune response [6].

Clinical features of TA vary according to the phase of the disease and the anatomical part that has been affected. The first phase is the systemic or pre pulseless phase during which patient may present with symptoms such as fever, malaise, night sweats, weight loss and anorexia, as was the case with our patient [7]. Second phase or the vasculitis phase features of vascular involvement such as tenderness over the vessels are noted in addition to the constitutional symptoms. In the final phase, also known as late or fibrotic phase, arterial stenosis leads to pulseless disease [7].

In addition to the constitutional symptoms, patients eventually present with symptoms of vessel occlusion or stenosis such as hypertension, pulse deficit, blood pressure differences between extremities, vascular bruits and claudication. Neurological features such as headache, cognitive dysfunction, seizures and rarely cerebrovascular accidents have been observed [8]. Cutaneous manifestations of TA include purpura, urticaria, erythema nodosum [9].

Till date, no specific laboratory biomarkers have been validated for diagnosis of TA. Acute phase reactants such as CRP and ESR are useful in monitoring disease progression.

In 1990, the American College of Rheumatology endorsed a classification criterion for Takayasu arteritis which has been modified in 2022 (Table 1) [10].

**Table 1:** 2022 American college of Rheumatology classification criteria for Takayasu arteritis. A score of ≥5 points is needed for the classification.

Classification criteria for Takayasu arteritis	
Absolute requirement	
Age ≤ 60 years at the time of diagnosis	
Evidence of vasculitis on imaging	
Additional clinical criteria	
Female sex	+1
Angina or ischemic cardiac pain	+2
Arm or leg claudication	+2
Vascular bruits	+2
Reduced pulse in upper extremity	+2
Carotid artery abnormality	+2
Systolic blood pressure difference in arms ≥20 mm Hg	+1
Additional imaging criteria	
Number of affected arteriole territories (select one)	
One arterial territory	+1
Two arterial territories	+2
Three or more arterial territories.	+3
Symmetric involvement of paired arteries	+1
Abdominal aorta involvement with renal or mesenteric involvement	+3

Imaging studies are essential for establishing the diagnosis of Takayasu arteritis and to determine the extent of vascular involvement. Patients with suspected case of Takayasu arteritis should undergo imaging of arterial tree of chest, abdomen, head and neck to look for luminal narrowing, occlusion or aneurysms. Conventional or CT angiography is necessary to evaluate the vessel lumen. In contrast to conventional angiography, CT angiography provides information on luminal diameter as well as arterial wall thickness. Ultrasonography of the carotid and subclavian artery provide complementary details of vascular wall, luminal diameter and blood flow in these vessels which may be commonly involved. Magnetic resonance angiography of the brain is useful to rule out vertebral vessel occlusion and ischemic changes.

In our case, CT angiography, Doppler ultrasonography of the carotid and vertebral arteries and CT of the brain were done which showed isolated aneurysm of the ascending aorta without steno occlusive lesions with normal subclavian and vertebral arteries along with a normal brain scan.

A study by Kumar et al., reported the incidence of aneurysm in TA to be 22.2 % [11]. Isolated aneurysm of the aorta as in our case is rare and is more often found in association with a steno-occlusive lesion elsewhere within the vessel [3,4]. Incidence of aortic regurgitation in TA is between 13 % and 25 % [12]. However cases of severe aortic insufficiency requiring surgical intervention, as in our case are much less common [4].

Histological features also vary in accordance with the phase of disease. Histologically, lesions maybe classified as active, chronic or healed [3]. In the active phase, inflammation starts at the junction of adventitia and medial layer around the vasa vasorum [3]. There is mononuclear cell infiltrate, necrosis, intimal proliferation, fibrosis and disruption of elastic layer [3]. Rapid, severe inflammation with loss of smooth muscles leads to formation of aneurysm. In the chronic phase there is patchy inflammation with fibrosis of medial layer and neovascularisation. The healed phase shows fibrosis only [3].

Medical intervention is the mainstay of treatment and aims to control inflammation of the arterial wall by use of corticosteroids as first line therapy. Biological agents such as TNF α inhibitors, tocilizumab and interleukin 6 inhibitors maybe used in refractory cases [6]. Indications for surgical intervention include severe aortic regurgitation, uncontrolled hypertension due to renal artery stenosis, aneurysm at risk of rupture and critical limb ischemia [13].

Aneurysmal aortitis in TA must be differentiated from other causes of aortic aneurysm such as tuberculosis or syphilis. Serological tests as well as histological features are helpful in differentiating these infective aetiologies. Giant cell arteritis maybe a differential however it commonly affects individuals >50 years of age with involvement of aorta being infrequent.

## 4 Conclusion

A diagnosis of Takayasu arteritis must be considered in all young patients with aneurysm of aorta with aortic insufficiency. A complete radiological evaluation of the aorta and its branches is necessary for diagnosis as well as disease monitoring. High degree of suspicion allows for early diagnosis and initiation of medical therapy leading to better survival outcomes. For cases complicated by uncontrolled hypertension, severe aortic insufficiency or aneurysm with impending rupture, prompt surgical management is necessary.

## Ethical approval

This study has been exempted from ethical approval by our institution.

## Sources of funding

There were no sponsors for this case report.

## CRediT authorship contribution statement

A.S.: Visualization, Writing-Original draft preparation. P.A.: Writing-Original draft preparation, Investigation

S.B.T.: Writing-Reviewing and editing and Supervision. B.K.C: Conceptualization

## Guarantor

Pritha Acharya

## Registration of research studies

Not applicable.

## Consent

Written informed consent was obtained from the patient for publication of this case report and accompanying images. A copy of the written consent is available for review by the Editor-in-Chief of this journal on request.

## Conflict of interest statement

There are no conflicts of interest in the creation of this case report as declared by the authors.
